# Interrelationship Between Craniocervical Dissociation Spectrum Injuries and Atlantoaxial Instability on Trauma Cervical MRI Examinations

**DOI:** 10.7759/cureus.31238

**Published:** 2022-11-08

**Authors:** Peter Fiester, Erik Soule, Jeet Patel, Matthew Jenson, Dinesh Rao

**Affiliations:** 1 Neuroradiology, University of Florida College of Medicine, Jacksonville, USA; 2 Interventional Radiology, University of Florida College of Medicine, Jacksonville, USA

**Keywords:** transverse atlantal ligament, craniocervical dissociation, atlantoaxial instability, magnetic resonance imaging, trauma

## Abstract

Background and purpose

Craniocervical dissociation injuries encompass a spectrum of osteoligamentous injuries between the skull base and C1-C2 that may be treated via prolonged external immobilization versus occipital cervical fusion depending on the risk of persistent craniocervical instability. However, the presence of atlantoaxial instability (AAI) at C1-C2, as determined by transverse atlantal ligament (TAL) integrity with or without a C1 fracture, may guide the neurosurgical management of craniocervical dissociation spectrum injuries (CDSI) since it implies an overall greater degree of instability at the craniocervical junction (CCJ).

Materials and methods

Adult trauma patients who suffered a transverse atlantal ligament injury on cervical magnetic resonance imaging (MRI) were identified retrospectively. The cervical computed tomography (CT) and magnetic resonance imaging examinations for these patients were reviewed for additional traumatic findings. Demographic information, treatment, and outcome information were recorded.

Results

Twenty-nine trauma patients presented to the emergency department (ED) with an acute, midsubstance transverse atlantal ligament tear on cervical magnetic resonance imaging. Thirty-one percent of patients demonstrated a tear in at least one major craniocervical ligament (atlanto-occipital capsular ligaments, alar ligaments, and tectorial membrane {TM}) with 14% demonstrating a tear in two major craniocervical ligaments and no patients demonstrating a tear in all three major craniocervical ligaments. Minor craniocervical ligament injuries (anterior atlanto-occipital membrane complex {AAOMc} and posterior atlanto-occipital membrane complex {PAOMc}) were common and observed in 76% of patients.

Conclusions

Our study suggests that multiple major craniocervical junction ligamentous injuries on cervical magnetic resonance imaging are relatively uncommon in the setting of transverse atlantal ligament injury.

## Introduction

Craniocervical dissociation spectrum injuries (CDSI) represent a spectrum of increasing hyperflexion-hyperextension forces applied to the osteoligamentous structures of the craniocervical junction (CCJ) that may range in severity from isolated craniocervical ligament tears to overt atlanto-occipital dislocation (AOD) [1,2]. Strictly defined, these injuries imply focal trauma between the structures responsible for maintaining integrity between the central skull base and C1-C2, namely, the atlanto-occipital joint, the major CCJ ligaments (capsular ligaments, alar ligaments, and tectorial membrane {TM}), the minor CCJ ligaments (anterior and posterior atlanto-occipital membranes), and the surrounding neck musculature (longus capitis muscle, obliquus capitis superior and inferior muscles, and rectus capitis posterior major and minor muscles). Additional named ligaments, including the apical ligament (vestigial notochordal remnant between the basion of the clivus and the C2 dens), the transverse occipital ligament, the accessory atlantoaxial ligament, and the superior band of the cruciform ligament, are not routinely visualized on cervical magnetic resonance imaging (MRI) and thought to play a nominal role in craniocervical stability [3,4].

The stabilizing structures of the CCJ proper are anatomically and physiologically distinct from the major stabilizing structure at C1-C2. In contrast to the other osteoligamentous CCJ structures, the transverse atlantal ligament (TAL) extends horizontally between the medial tubercles of the C1 lateral masses, extending posterior to the C2 dens, and locks the median atlantoaxial joint in place [5]. There is no direct attachment between the TAL and the skull base. Biomechanical cadaveric studies have demonstrated the unique functional role of the TAL as both a primary stabilizer of the atlantoaxial joint and a primary facilitator of atlantoaxial rotation at C1-C2 [6,7]. Secondary atlantoaxial stabilizers including the lateral atlantoaxial joints, the anterior atlanto-occipital membrane complex (AAOMc), and the longus capitis muscle also support the atlantoaxial joint.

The complex interplay between the craniocervical junction, including the atlanto-occipital joint and its surrounding supportive ligaments and muscles, and the atlantoaxial joint with its strong, stabilizing transverse atlantal ligament is not well understood. Unstable traumatic injuries of the atlanto-occipital and atlantoaxial joint often occur in isolation [8]. Regardless, the conventional teaching of the CCJ anatomy and biomechanics includes the atlantoaxial joint with the CCJ proper, although the anatomy, biomechanics, and traumatic injuries that occur between these two osteoligamentous structures are quite different. The neurosurgical literature regarding the clinical management of CDSI includes conflicting data on the appropriateness of occipital cervical fusion and fusion length/level versus prolonged external immobilization. While occipital cervical fusion is necessary for overt atlanto-occipital dislocation, craniocervical injuries with intact atlanto-occipital joints may be treated with prolonged external immobilization. In contrast, atlantoaxial instability (AAI) is typically treated with operatively via posterior C1-C2 fusion or anterior C1 osteosynthesis [9]. The challenge is the appropriate balance between preserving function and the range of motion with the risk of persistent instability and spinal cord injury. The confusion partly stems from whether CCJ instability involves the atlanto-occipital joint, the atlantoaxial joint, or both. The lack of consensus regarding the neurosurgical management of CDSI is complicated by the high morbidity and mortality of these injuries, which often limits operative management. The incomplete understanding of the association between CDSI and atlantoaxial instability (AAI) injuries on cervical MRI in the radiology literature may also play a role [10-12].

Therefore, the purpose of our study is to identify adult trauma patients with a confirmed acute, post-traumatic tear of the transverse atlantal ligament on cervical MRI and evaluate for concomitant craniocervical junction ligamentous injuries. We hypothesize that atlantoaxial instability more commonly occurs in isolation from atlanto-occipital instability on cervical MRI. A thorough understanding and discussion of the complex interplay between instability at the CCJ proper and the atlantoaxial joint may allow for improved consensus regarding operative management of these injuries.

## Materials and methods

A waiver of informed consent was granted by the institutional review board (IRB) chairman to retrospectively evaluate the imaging and clinical findings of adult trauma patients (>18 years old) with TAL injuries. Thirty patients who presented to the emergency department (ED) with an intrasubstance tear of the TAL on cervical MRI were identified retrospectively by using the keywords “transverse ligament,” “transverse band,” “cruciform ligament,” or “transverse atlantal ligament” included in cervical MRI reports between January 2015 and January 2021 using Nuance mPower (Nuance Communications, Burlington, MA) software. As a level I trauma center and tertiary care trauma spine center that captures all high-speed motor vehicle accidents (MVA) for a large geographic region, we were able to perform an analytic search of more than 15,000 cervical MRI examinations that presented to the ED for trauma during this time frame. The evaluation of the major and minor CCJ ligaments was performed, including the evaluation of the atlanto-occipital capsular ligaments, alar ligaments, tectorial membrane, and anterior and posterior atlanto-occipital membranes. Bony fractures of the craniocervical junction on preceding cervical spine computed tomography (CT) in addition to evaluation for intracranial trauma and cervical cord injury were recorded. Neurosurgical management and clinical outcomes for these injuries were analyzed.

Patient selection

Inclusion criteria for our patient cohort included all adult trauma patients who presented to our level I trauma center with a high-velocity injury with a confirmed TAL tear on cervical MRI. Exclusion criteria included pediatric patients less than 16 years old, non-trauma patients, and patients without a preceding cervical spine CT. Medial tubercle avulsion fracture of C1 without a midsubstance TAL tear was also excluded from the study. Patients with excessive inflammatory pannus or osteoarthritis of the atlantoaxial joint on cervical MRI that obscured the TAL and CCJ ligaments were also excluded from the study. The confirmation of a TAL tear was agreed upon in consensus by two certificate of additional qualification (CAQ)-certified neuroradiologists with long-term experience and prior research in craniocervical trauma.

Imaging protocols

CT and MRI examinations were performed using the standard departmental protocols. CT images were generated with 0.625 mm slice thickness and reconstructed using multiplanar bone and soft tissue algorithms (GE Medical Systems). Evaluation for CCJ bony trauma utilized multiplanar, orthogonal reconstructions using a thin-section bone kernel window. MRI studies were performed on a 1.5 Tesla magnet with a head and neck coil (Avanto, Siemens, Munich, Germany). Slice thickness was 3 mm, and sagittal T1, T2, and short tau inversion recovery (STIR), as well as axial T2 and T2 multi-echo data image combination (MEDIC) sequences, were obtained. All MRI sequences were comprehensively reviewed, and TAL injury location and type were agreed upon in consensus.

MRI criteria for injury

All cervical MRI examinations were graded as non-diagnostic, limited, or diagnostic quality. TAL injuries were confirmed primarily on T2 and STIR axial and sagittal MRI sequences when there was clear disruption of the normal dark T2 hypointense ligament posterior to the C2 dens and increased T2 signal within the ligament. The TAL was also considered injured if the ligament was not visible and replaced with hematoma (ruptured ligament). The location of the TAL injury was recorded as right, left, midline, or bilateral in relation to the C2 dens. Injury of the capsular ligaments was inferred on cervical CT and MRI as widening of the atlanto-occipital joint greater than 3 mm. Alar ligament injury was directly evaluated on axial and sagittal T2-weighted sequences as increased T2 signal and focal disruption of the normal T2 hypointense band. Tectorial membrane (TM) injuries were confirmed primarily on T2 and STIR axial and sagittal imaging according to the classification system developed by Fiester et al. when there was disruption of the normal dark T2 hypointense ligament on cervical MRI and increased T2 signal within the ligament (type 2 tear) or the TM demonstrated increased T2 signal with greater than 50% decrease in TM thickness on sagittal T2-weighted imaging (type 3 tear). Anterior and posterior atlanto-occipital membrane tears were considered injured on axial and sagittal T2 and STIR sequences when there was clear disruption of the ligament and/or increased STIR signal within the ligament.

Supplemental data

Electronic patient records were reviewed for the following: 1) age and sex of patient, 2) mechanism of trauma, 3) management (e.g., surgery, external fixation, and cervical collar), and 4) clinical outcome (e.g., ambulatory, wheelchair, and paretic). A good clinical outcome/full recovery was defined as the absence of persistent neurologic deficits on follow-up evaluation by the neurosurgery department at least four months following the initial injury.

## Results

A total of 29 (17 males and 12 females) patients were identified with TAL tears on cervical MRI between January 2015 and January 2021. Thirteen TAL tears were left-sided, 12 injuries were right-sided, two injuries were bilateral, and two injuries were midline (Table 1).

**Table 1:** Locations of traumatic tears of the transverse atlantal ligament, with demographic information and additional osteoligamentous trauma. AAOMc: anterior atlanto-occipital membrane complex; PAOMc: posterior atlanto-occipital membrane complex; TM: tectorial membrane; MVA: motor vehicle accident; M: male; F: female; TAL: transverse atlantal ligament; CCJ: craniocervical junction

Number	Sex/age	Mechanism of injury	TAL tear	CCJ ligamentous injury	C1-C2 fracture
1	M/37 years	MVA	Left	AAOMC; PAOMc	C1
2	F/73 years	MVA	Left	AAOMc; type 2 TM	C1
3	M/48 years	MVA	Right	AAOMc; right alar; type 2 TM	C2 transverse process
4	F/18 years	Pedestrian versus automobile	Right	PAOMc	None
5	M/77 years	MVA	Right	AAOMc; right alar; PAOMc	C1
6	M/32 years	MVA	Left	PAOMc	C1
7	F/30 years	Pedestrian versus automobile	Right	AAOMc; PAOMc	C1
8	M/85 years	MVA	Bilateral	AAOMc; PAOMc	C1
9	F/39 years	MVA	Left	AAOMc; PAOMc	C1; type 2 dens
10	F/35 years	MVA	Left	AAOMc; PAOMc	C1; C2 pars
11	M/69 years	Pedestrian versus automobile	Midline	None	None
12	M/61 years	MVA	Left	None	Type 3 dens
13	F/59 years	MVA	Left	None	Type 2 dens
14	M/56 years	MVA	Left	PAOMc	Type 3 dens
15	M/27 years	MVA	Left	PAOMc	None
16	M/26 years	MVA	Right	Right alar; PAOMc	None
17	M/24 years	MVA	Right	PAOMc	None
18	F/18 years	Cyclist versus automobile	Right	None	None
19	F/33 years	MVA	Left	Type 2 TM; PAOMc	None
20	F/18 years	MVA	Right	None	None
21	M/68 years	MVA	Right	None	C2 pars interarticularis
22	M/19 years	MVA	Right	PAOMc	None
23	M/30 years	MVA	Left	PAOMc	None
24	F/62 years	MVA	Bilateral	AAOMc; bilateral alar; type 2 TM; PAOMc	None
25	F/35 years	Cyclist versus automobile	Left	None	C2 pars interarticularis
26	M/48 years	MVA	Midline	AAOMc; right alar; type 2 TM; PAOMc	None
27	M/27 years	Cyclist versus automobile	Left	AAOMc; left alar; PAOMc	C1
28	F/35 years	MVA	Right	AAOMc; bilateral alar; type 2 TM; PAOMc	C1; type 2 dens
29	M/69 years	MVA	Right	AAOMc	C1

**Figure 1:**
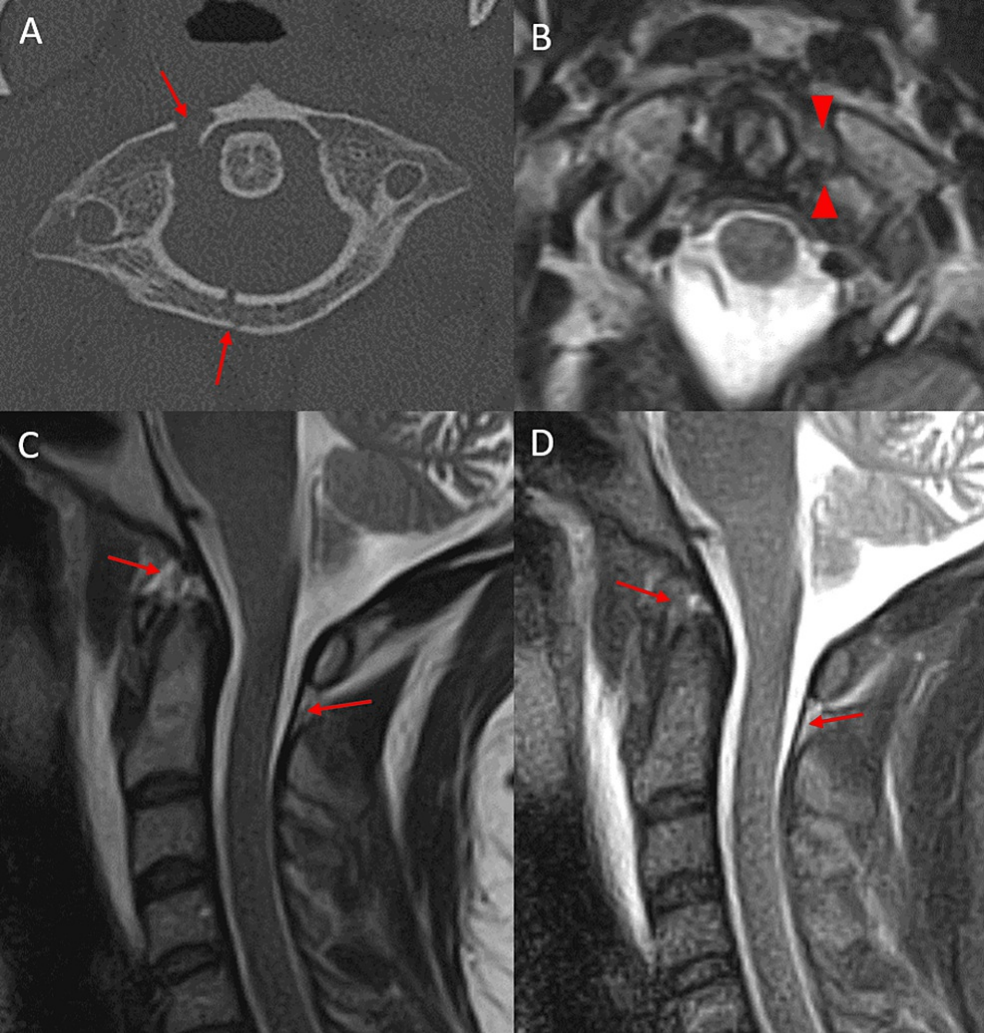
Axial CT of the cervical spine (A) in a 30-year-old trauma patient status post MVA demonstrating C1 anterior and posterior arch fractures (red arrows) and right lateral mass fracture. Axial T2-weighted MRI (B) in the same patient demonstrating a left transverse atlantal ligament tear (red arrowheads). Sagittal T2-weighted (C) and STIR-weighted (D) MRI of the cervical spine in the same patient demonstrating low-grade anterior and posterior atlanto-occipital membrane complex injuries (red arrows) with otherwise intact major craniocervical junction ligaments. Anterior and posterior atlanto-occipital membrane complex tears were relatively common in the setting of transverse atlantal ligament tears. CT: computed tomography; MVA: motor vehicle accident; MRI: magnetic resonance imaging; STIR: short tau inversion recovery

Thirty-one percent of patients demonstrated a tear in at least one major craniocervical ligament (atlanto-occipital capsular ligaments, alar ligaments, and tectorial membrane), 14% of patients demonstrated a tear in two or more major craniocervical ligaments, and no patients demonstrated a tear in all three major ligaments. Increased STIR signal and/or frank disruption involving at least one minor craniocervical ligament was more common and present in 76% of patients (Figure 1A-1D).

**Figure 2:**
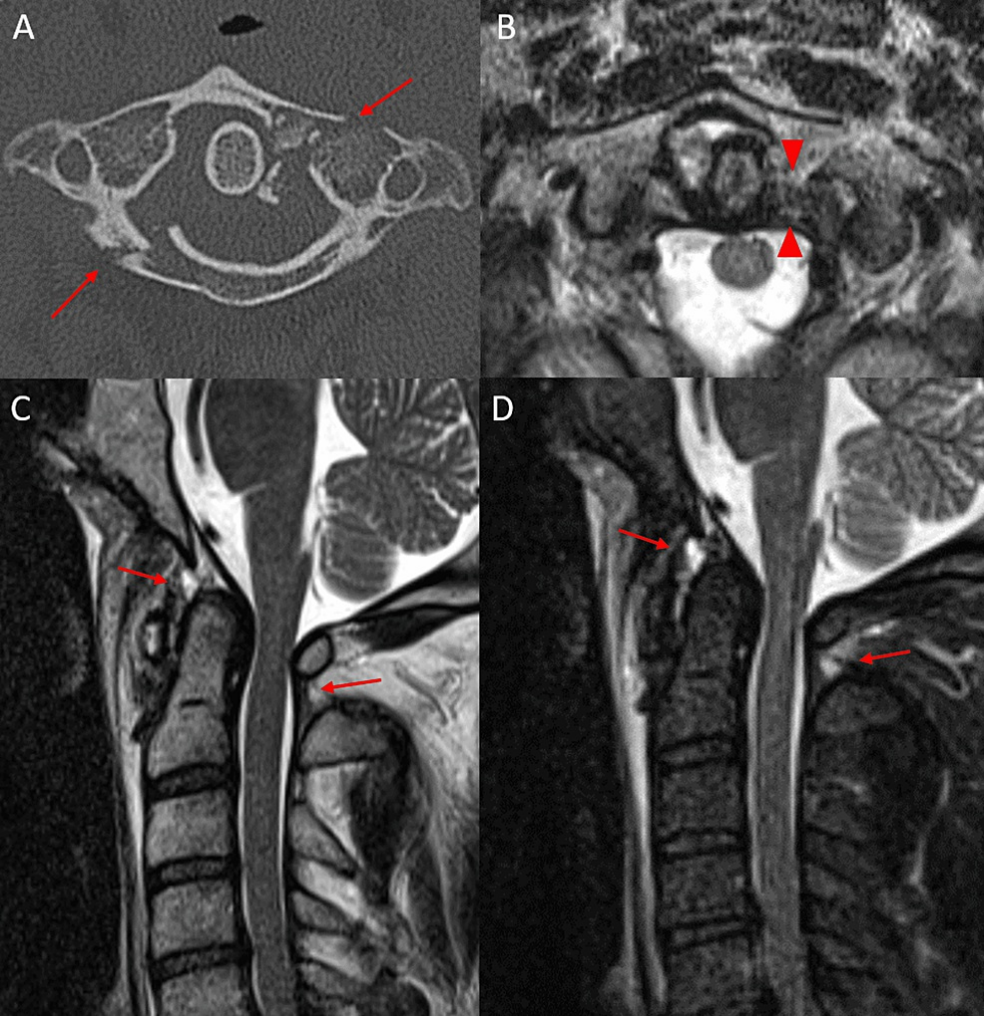
Axial CT of the cervical spine (A) in a 32-year-old trauma patient status post MVA demonstrating comminuted, left C1 anterior and posterior arch fractures (red arrows). Axial T2-weighted MRI (B) in the same patient demonstrating a left transverse atlantal ligament tear (red arrowheads). Sagittal T2-weighted (C) and STIR-weighted (D) MRI of the cervical spine in the same patient demonstrating low-grade anterior and posterior atlanto-occipital membrane complex injuries (red arrows) with otherwise intact major craniocervical junction ligaments. Anterior and posterior atlanto-occipital membrane complex tears were relatively common in the setting of transverse atlantal ligament tears. CT: computed tomography; MVA: motor vehicle accident; MRI: magnetic resonance imaging; STIR: short tau inversion recovery

A total of 14 patients demonstrated an anterior atlanto-occipital membrane injury, 20 patients demonstrated a posterior atlanto-occipital membrane complex (PAOMc) injury, and 13 patients demonstrated an injury in both minor ligaments (Figure 2A-2D).

Fifty-nine percent of patients demonstrated a C1 and/or C2 fracture (10 patients with C1 burst fractures and eight patients with type II/III dens fracture). Seventy-six percent of patients demonstrated intracranial hemorrhage on concurrent head CT. There was zero case of cervical cord contusion. All TAL injuries were the result of an MVA, either a primary MVA (25 patients) or a pedestrian/bicyclist struck by a motor vehicle (four patients). Surgical management varied, depending on the extent of the CCJ injury, patient comorbidities, and other post-traumatic injuries at the time of the trauma. Two patients underwent occipital cervical fusion (both occiput-C3), four patients underwent occipital-sparing posterior cervical fusion (three patients with C1-C2 fusion and one patient with C1-C6 fusion), two patients underwent prolonged external immobilization with halo bracing, and the remaining patients were treated with Miami J (Össur, Reykjavík, Iceland) collar placement. Seven patients experienced persistent neurologic deficits (weakness and/or spasticity) at least four months following the initial trauma.

## Discussion

**Figure 3:**
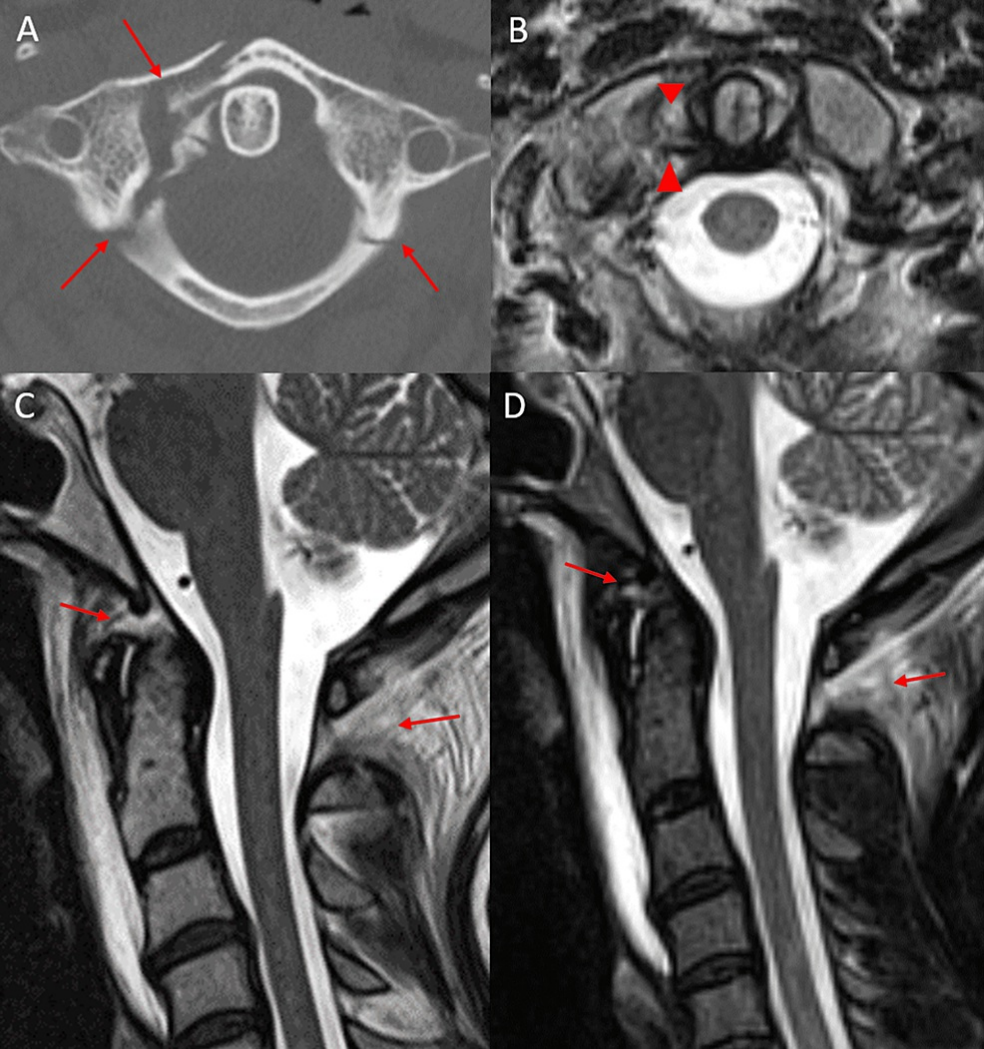
Axial CT of the cervical spine (A) in an 18-year-old trauma patient status post MVA demonstrating comminuted, right C1 anterior arch fracture and bilateral posterior arch fractures (red arrows). Axial T2-weighted MRI (B) in the same patient demonstrating a right transverse atlantal ligament tear (red arrowheads). Sagittal T2-weighted (C) and STIR-weighted (D) MRI of the cervical spine in the same patient demonstrating anterior and posterior atlanto-occipital membrane complex injuries (red arrows) with otherwise intact major craniocervical junction ligaments. Anterior and posterior atlanto-occipital membrane complex tears were relatively common in the setting of transverse atlantal ligament tears. CT: computed tomography; MVA: motor vehicle accident; MRI: magnetic resonance imaging; STIR: short tau inversion recovery

Despite their distinct anatomic and biomechanical properties and differences in the types of post-traumatic injuries sustained, the interrelation between atlantoaxial instability and the instability of the CCJ proper is poorly understood. This negatively impacts clinical management and has hindered the ability to reach a consensus regarding appropriate neurosurgical care. Our study demonstrates that a minority of patients with atlantoaxial instability (defined by a midsubstance TAL injury) had concomitant major CCJ ligamentous injury with only 14% of patients demonstrating an additional injury of at least two major CCJ ligaments and zero patient demonstrating a tear of all three major CCJ proper ligaments. Moreover, no patients exhibited imaging findings of overt atlanto-occipital dislocation, typically defined as atlanto-occipital joint space widening greater than 2 mm. Minor CCJ ligament injuries involving the anterior atlanto-occipital membrane complex (AAOMc) or posterior atlanto-occipital membrane complex (PAOMc) were more common and present in 76% of patients (Figure 3A-3D).

However, Chang et al. recently investigated the utility of isolated increased STIR signal in the PAOMc on MRI as a secondary finding of C1-C2 fractures with a high sensitivity (89.7%) [13]. Given that 59% of our patient cohort exhibited a C1-C2 fracture, increased STIR signal may have been a secondary finding related to a bony fracture rather than a primary ligamentous injury of the AAOMc and PAOMc.

As injuries to the atlantoaxial joint and CCJ proper more commonly occur in isolation, redefining the radiologic and neurosurgical definition of the CCJ by separating the CCJ proper (defined as the atlanto-ooccipital joint and surrounding supportive ligaments between the skull base and C1-C2) from the atlantoaxial joint (defined as the median and lateral atlantoaxial joint and TAL) would have several positive effects. With the increasing availability and utilization of cervical MRI in the ED trauma setting, the radiologist now has the opportunity to accurately define CDSI by highlighting injuries to the specific major and minor CCJ ligaments and TAL [14]. Understanding the unique biomechanics and contributions of each ligament toward maintaining CCJ stability would help guide the neurosurgeon in determining which CDSI injury patterns predispose to instability at the CCJ proper (treated with occipital cervical fusion), the atlantoaxial joint (treated with occipital-sparing fusion), or both. Reducing the number of patients who undergo post-operative fusion or reducing fusion length would significantly improve the range of motion and increase the patient’s quality of life. Several studies have demonstrated the risks associated with occipital surgical fusion in addition to the reduction in quality of life and restricted mobility [15-18]. Often, the clinical decision to perform occipital-cervical fusion in patients with CDSI is made based on the potential for future CCJ instability; however, there is no consensus in the neurosurgical literature to define which CDSI patterns actually predispose to instability at the CCJ. This is even more problematic as “occult AOD” is being more commonly observed in patients with little or no atlanto-occipital joint space widening on cervical CT but with major CCJ ligament tears on follow-up cervical MRI.

CDSI encompasses a spectrum of osteoligamentous injuries at the CCJ and atlantoaxial joint that range from isolated CCJ proper major or minor ligamentous injuries to overt AOD with atlanto-occipital dislocation. In our patient cohort, injuries to the atlantoaxial joint were much more commonly isolated injuries without injury of the major and minor CCJ proper ligaments. Many trauma centers employ a high-resolution isotropic sequence as part of their trauma cervical MRI protocols that allow multiplanar, three-dimensional reconstructions and improved anatomic resolution of the CCJ ligaments and TAL. Along with specialized isotropic imaging, increasing magnet field strength allows improved anatomic resolution of the complex osteoligamentous anatomy of the CCJ [19]. These developments aid radiologists in determining the specific integrity of the individual major and minor CCJ ligaments and TAL in high-velocity trauma patients to help guide neurosurgical management and aid in future research.

**Figure 4:**
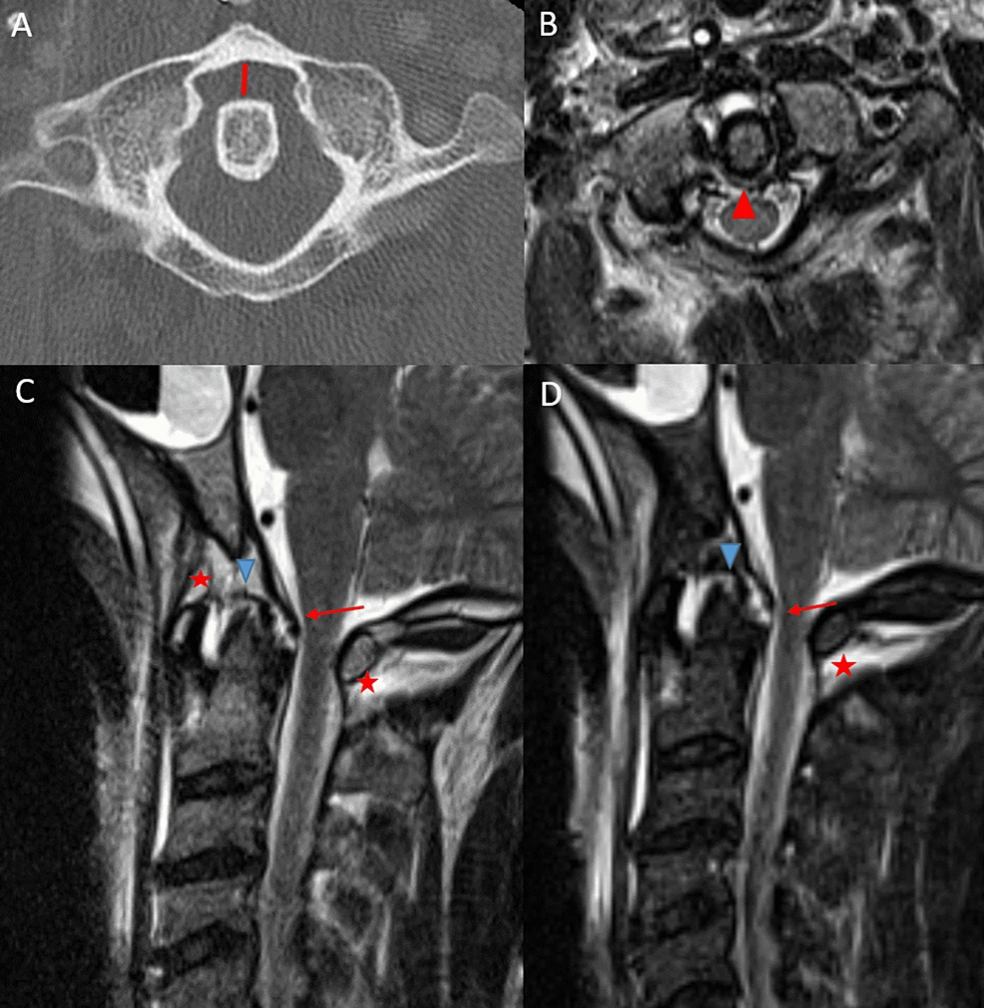
Axial CT of the cervical spine (A) in a 48-year-old trauma patient status post MVA demonstrating widening of the anterior atlanto-dens interval (red line) without a C1 fracture. Axial T2-weighted MRI (B) in the same patient demonstrating a central transverse atlantal ligament tear (red arrow). Sagittal T2-weighted (C) and STIR-weighted (D) MRI of the cervical spine in the same patient demonstrating subclival tectorial membrane tear (red arrows), right alar ligament tear (blue arrowheads), and anterior and posterior atlanto-occipital membrane complex tears (red stars). The majority of observed patients in our study with atlantoaxial instability did not have major craniocervical junction ligament tears. CT: computed tomography; MVA: motor vehicle accident; MRI: magnetic resonance imaging; STIR: short tau inversion recovery

In addition to recognizing the spectrum of post-traumatic imaging appearances of CDSI, understanding the unique biomechanical role of each ligament in maintaining CCJ and atlantoaxial integrity is necessary to optimize clinical management. Regarding the CCJ proper, three major ligaments maintain CCJ integrity: the atlanto-occipital joint and enveloping capsular ligament, the bilateral alar ligaments, and the tectorial membrane [4,5]. Injuries to one or more of these major ligaments imply a greater risk for craniocervical instability and higher likelihood for occipital cervical fusion (Figure 4A-4D).

Fiester et al. identified nine patients who underwent emergent occipital cervical fusion for acute CDSI and found that all nine patients demonstrated a tear in the tectorial membrane and five patients sustained an alar ligament tear [20]. The importance of the major CCJ ligaments is supported from cadaveric studies that have investigated the in vitro biomechanical function of these three ligaments [21]. In contrast, the minor CCJ proper ligaments, namely, the AAOMc and PAOMc, are thought to play a nominal role in maintaining CCJ integrity. Increased STIR signal within the PAOMc is not uncommonly seen in the setting of a C1-C2 fracture as opposed to a primary PAOMc ligamentous injury [4,5,13].

In contrast to the numerous contributory ligaments for CCJ stability, biomechanical research studies indicate that the TAL acts as the primary stabilizer of the atlantoaxial joint and the sole preventer of anterior subluxation of C1 on C2 [6,7]. Radiologically, TAL injuries are traditionally inferred by the widening of the anterior atlanto-dens interval (ADI) greater than 2 mm on cervical spine CT and plain film. Multiple large research studies have established normative values for the anterior ADI in pediatric and adult patients [22-26]. However, in practice, the notion of the TAL as the primary stabilizer of the atlantoaxial joint is being further scrutinized. Secondary stabilizers, including the facet capsule of the lateral atlantoaxial joint and surrounding neck musculature, may help restore anatomic congruence and play a role in stabilizing the C1-C2 joint in addition to the TAL. This concept is supported by current research studies in the neurosurgical literature that TAL disruption does not inherently lead to C1-C2 instability in the setting of a C1 burst fracture [27-29]. In fact, TAL-deficient C1 burst fractures have been treated more conservatively with either anterior C1 osteosynthesis or external immobilization with excellent clinical outcomes, thus challenging the long-held assertion by Dickman et al. that a TAL disruption in the setting of a C1 fracture necessitates prompt C1-C2 fusion [30].

Consensus regarding the appropriateness and necessity of operative fusion and length versus conservative management for CDSI is debated in the neurosurgical literature especially in the absence of overt AOD, which, if survivable, is almost always treated with prompt surgical stabilization. However, the patient’s age and comorbidities play a major role in the overall morbidity and mortality of craniocervical and atlantoaxial trauma [11]. One recent study demonstrated wide variability in the types of osteoligamentous trauma and the number of torn CCJ ligaments in patients who underwent occipital cervical fusion for CDSI [20]. Furthermore, there has been wide variability in the surgical techniques and clinical management of patients with CDSI implying that further research and/or a more nuanced understanding of CDSI is warranted to improve patient care. Separating and thoroughly understanding the distinct anatomic and biomechanical properties of the CCJ proper from the atlantoaxial joint would be a beneficial first step for the clinical management, optimization, and future investigative research of CDSI.

The limitations of our study were subject to those of a retrospective review and included the possibility of reinforcement bias and interpreter error. However, image quality on cervical MRI was graded as diagnostic in all cases to identify a TAL injury. Then, by nature of a retrospective review, we may not have captured all TAL injuries and did not specifically identify C1 medial tubercle avulsion fractures; however, this was deliberate since we wanted to concentrate solely on patients with midsubstance TAL tears. Finally, our results are limited from the small sample size, and although we collected one of the largest cohorts of midsubstance TAL injuries on cervical MRI in the literature, a multiregional research study with a larger sample size investigating the relationship of CCJ and atlantoaxial trauma in CDSI would improve the accuracy of our findings and assist in targeted research studies comparing operative to non-operative management in the patients with different CDSI injury types on cervical CT and MRI.

## Conclusions

CDSI is a spectrum of increasing, post-traumatic hyperflexion-hyperextension forces applied to the osteoligamentous CCJ and encompasses both the anatomy and biomechanics of the CCJ proper and the atlantoaxial joint. Given the variability and range of imaging findings on CT and MRI, significant controversy exists in the literature regarding the proper clinical management of patients with traumatic CDSI. Our study suggests that major CCJ proper ligamentous injury on cervical MRI is a relatively uncommon finding in the setting of transverse atlantal ligament injury. A thorough understanding and separation of the distinct anatomic and biomechanical properties of the CCJ proper from the atlantoaxial joint would be beneficial to optimize clinical management and guide future investigative research of craniocervical and upper cervical spine trauma.
